# Diverse application platform for hard X-ray diffraction in SACLA (DAPHNIS): application to serial protein crystallography using an X-ray free-electron laser

**DOI:** 10.1107/S1600577515004464

**Published:** 2015-04-16

**Authors:** Kensuke Tono, Eriko Nango, Michihiro Sugahara, Changyong Song, Jaehyun Park, Tomoyuki Tanaka, Rie Tanaka, Yasumasa Joti, Takashi Kameshima, Shun Ono, Takaki Hatsui, Eiichi Mizohata, Mamoru Suzuki, Tatsuro Shimamura, Yoshiki Tanaka, So Iwata, Makina Yabashi

**Affiliations:** a Japan Synchrotron Radiation Research Institute, 1-1-1 Kouto, Sayo-cho, Sayo-gun 679-5198, Japan; b RIKEN SPring-8 Center, 1-1-1 Kouto, Sayo-cho, Sayo-gun 679-5148, Japan; c Department of Applied Chemistry, Graduate School of Engineering, Osaka University, 2-1 Yamadaoka, Suita, Osaka 565-0871, Japan; d Institute for Protein Research, Osaka University, 3-2 Yamadaoka, Suita, Osaka 565-0871, Japan; e Department of Cell Biology, Graduate School of Medicine, Kyoto University, Yoshidakonoe-cho, Sakyo-ku, Kyoto 606-8501, Japan

**Keywords:** serial femtosecond crystallography, XFEL

## Abstract

An experimental system for serial femtosecond crystallography using an X-ray free-electron laser (XFEL) has been developed. It basically consists of a sample chamber, fluid injectors and a two-dimensional detector. The chamber and the injectors are operated under helium atmosphere at 1 atm. The ambient pressure operation facilitates applications to fluid samples. Three kinds of injectors are employed to feed randomly oriented crystals in aqueous solution or highly viscous fluid. Experiments on lysozyme crystals were performed by using the 10 keV XFEL of the SPring-8 Angstrom Compact free-electron LAser (SACLA). The structure of model protein lysozyme from 1 µm crystals at a resolution of 2.4 Å was obtained.

## Abstract 2

An experimental platform for serial femtosecond crystallography using an X-ray free-electron laser and its applications at SACLA are described.

## 1. Introduction   

An X-ray free-electron laser (XFEL) offers opportunities for high-resolution structure analysis using X-ray pulses with a large number of photons and a short duration (Emma *et al.*, 2010 ▸; Ishikawa *et al.*, 2012 ▸). The SPring-8 Angstrom Compact free-electron LAser (SACLA) provides ∼10^11^ photons in a pulse with a duration shorter than 10 fs. These characteristics are advantageous in application for protein crystallography, as even a single XFEL pulse can provide enough photons for recording a diffraction pattern from a protein crystal. The ultrashort pulse nature allows circumventing radiation damage on the sample because diffraction events can be terminated within a time scale much shorter than that of the damage process (Barty *et al.*, 2012 ▸).

Serial femtosecond crystallography (SFX) has been developed at the Linac Coherent Light Source (LCLS) for recording a considerable number of damage-free diffraction patterns from tiny crystals (Chapman *et al.*, 2011 ▸; Boutet *et al.*, 2012 ▸). This method often adopts a fluid carrier to continuously feed crystals of micrometer sizes and with random orientations. The stream of crystals in the fluid interacts with the XFEL beam which is generally focused to have a spot size comparable with the crystal size. High photon density in the focused beam enables single-pulse diffraction within femtosecond exposure. Recent publications show the successful application of SFX at LCLS (Kern *et al.*, 2012 ▸, 2013 ▸; Redecke *et al.*, 2013 ▸; Liu *et al.*, 2013 ▸; Demirci *et al.*, 2013 ▸; Barends *et al.*, 2014 ▸).

The usefulness of SFX has stimulated us to develop a measurement system at SACLA. We constructed a prototypical system for SFX by modifying instruments for coherent X-ray diffraction imaging (CXDI) (Miao *et al.*, 1999 ▸). This system basically consists of a 1 µm focusing system with Kirkpatrick–Baez (KB) mirrors (Yumoto *et al.*, 2013 ▸), a multiple application X-ray imaging chamber (MAXIC) (Song *et al.*, 2014 ▸), a liquid-jet injector and a multi-port charge-coupled device (MPCCD) detector (Kameshima *et al.*, 2014 ▸). The feasibility of SFX at SACLA was demonstrated by applying the prototype to lysozyme crystals (Song *et al.*, 2014 ▸).

The MAXIC in the prototypical system is an in-vacuum diffractometer which is originally dedicated for CXDI and has compatibility with SFX. From a practical point of view, however, the dual functionality of the MAXIC is not necessarily useful for SFX. Although the in-vacuum operation allows us to make background signals as small as possible, it can cause serious problems with the sample injection. In particular, the vacuum environment accidentally makes sample liquids freeze. This could hinder the stable operation of liquid injectors and, more seriously, intense diffracted X-rays from a frozen sample may leave serious damage on a detector. We also expect to have difficulty with the MAXIC in injecting protein crystals in a lipidic cubic phase (LCP) matrix, especially in a monooleoin LCP matrix, which is commonly used in the LCP crystallization technique. Injection into the vacuum chamber could cause the transition of a monoolein LCP to a crystalline phase which produces unwanted diffraction patterns (Weierstall *et al.*, 2014 ▸). Although the phase transition is avoidable by using another LCP host lipid or adding a different kind of lipid to a host monoolein (Liu *et al.*, 2014 ▸), the sample preparation method could become simpler by being free from additives or limitations on host lipids.

On the basis of the knowledge obtained from the test experiment, we have developed an independent SFX system to realise efficient operation by uncoupling the dual functionality. The system allows us to keep a sample under atmospheric pressure during measurement. The ambient-pressure operation prevents the sample from freezing and facilitates the control of temperature and humidity around the sample. These advantages make the system widely applicable beyond protein crystals, *e.g.* live organisms, solutions and powders dispersed in liquids.

In this paper we describe the design and performance of the experimental platform DAPHNIS (Diverse Application Platform for Hard X-ray diffractioN In SACLA), which is dedicated for SFX at SACLA in order to facilitate the measurement of a wide variety of samples. SFX experiments with this system have been performed using lysozyme crystals. Results of the experiments are shown.

## 2. Design   

### 2.1. Overview of the whole system   

**Figure 1:**
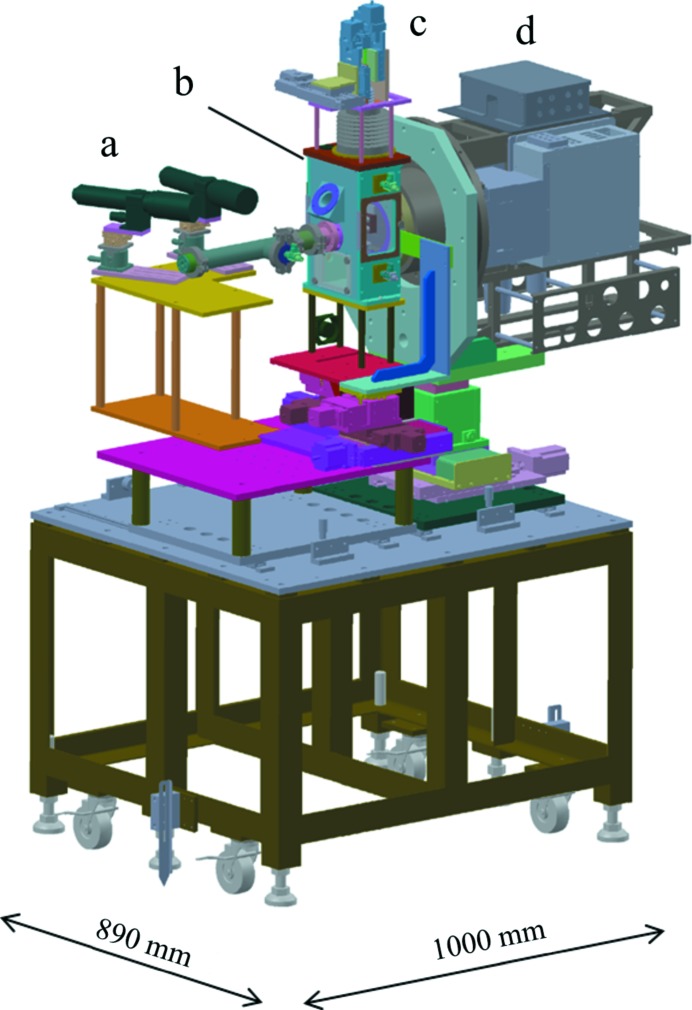
The DAPHNIS system: (*a*) microscopes for sample monitoring, (*b*) sample chamber, (*c*) injector manipulator and (*d*) the MPCCD detector.

Fig. 1 ▸ shows a drawing of the whole system of DAPHNIS which primarily consists of a sample chamber, injectors and an MPCCD detector with eight sensor modules. This system is usually combined with the 1 µm focusing system. An XFEL beam from the focusing system enters the sample chamber through a beryllium window. Samples are delivered to the focal point with sample injectors which are mounted on a motorized manipulator. The XFEL beam is blocked by a beam stopper. Diffracted X-rays are detected with the MPCCD detector on a shot-to-shot basis. The distance between the MPCCD sensor and the sample is adjustable in the range between 50 and 100 mm. A nominal resolution range of the current system is 37–1.5 Å (37–2.5 Å) at a sensor-to-sample distance of 50 mm (100 mm) and an X-ray wavelength of 1.24 Å. The upper and lower limits are determined by the beam-stopper diameter and the sensor-area span (see §2.2 and §2.4).

### 2.2. Sample chamber   

**Figure 2:**
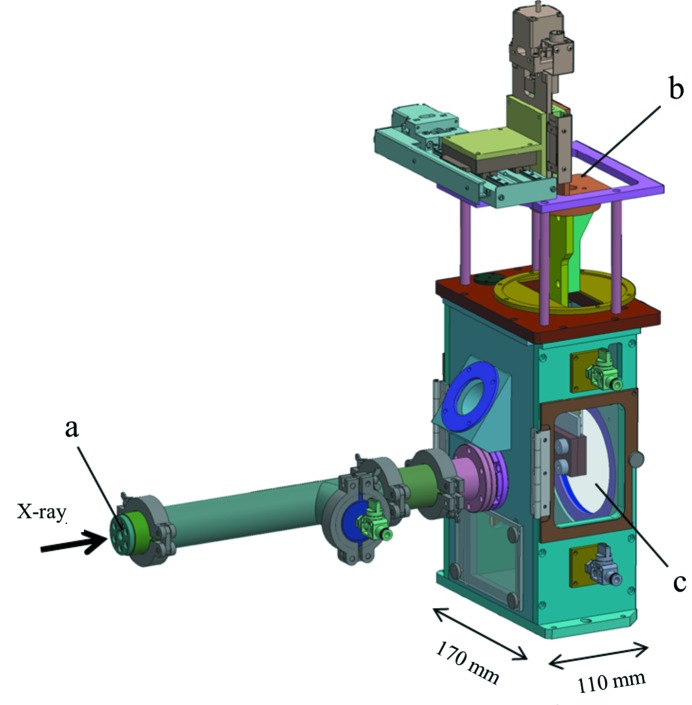
Sample chamber in DAPHNIS: (*a*) beryllium window for XFEL, (*b*) injector holder and (*c*) polyimide window for diffracted X-rays.

Fig. 2 ▸ shows a drawing of the sample chamber, which is equipped with a beam-inlet beryllium window, a polyimide window for diffracted X-rays, a pinhole, a beam stopper and an injector manipulator. The main body has a rectangular shape of dimensions 190 mm × 330 mm × 113 mm. It is mounted on motorized translation stages for alignment to the XFEL beam. Two microscopes are situated outside the chamber to monitor the sample at the interaction point. The chamber can be filled with helium gas at 1 atm, so that absorption and scattering of X-rays by air can be suppressed. The concentration of helium reaches 98% in 5 min at a flow rate of ∼2 l min^−1^. Although the 98% helium slightly raises the background level of a diffraction image, it makes only a minor contribution. The background mainly originates from scattered X-rays from the carrier fluid. For example, a grease carrier typically produces an intense circular diffraction pattern in the range <0.3 Å^−1^ and a diffuse scattering pattern, of which radially averaged intensities are greater than two photons per pixel in the range 0.3–0.5 Å^−1^ and one to two photons per pixel for 0.5–0.8 Å^−1^ at an X-ray wavelength of 1 Å and a sample-to-sensor distance of 55 mm. On the other hand, helium background intensities are less than one photon per pixel in the range 0.3–0.8 Å^−1^.

#### 2.2.1. X-ray windows   

For the beam inlet window, we adopt a beryllium foil with speckle-free quality to avoid the deterioration of the coherent wavefront of the XFEL (Goto *et al.*, 2007 ▸). The window has a thickness of 0.05 mm and an effective diameter of 10 mm. The distance between the window and the interaction point is 0.4 m, long enough to avoid damage induced by a focused XFEL beam from the KB mirrors with focal lengths of 2 m (vertical direction) and 1.55 m (horizontal direction).

The rear window for diffracted X-rays has a circular opening with a 117 mm diameter. The window material is a polyimide film of 0.05 mm thickness.

#### 2.2.2. Pinhole   

The pinhole with a diameter of 1 mm is situated between the beam inlet window and the interaction point to block off stray X-rays, which are mainly scattered from the focusing optics, the air section and the X-ray windows. The distance between the pinhole and the inter­action point is 50 mm.

#### 2.2.3. Beam stopper   

The beam stopper is a 1 mm-diameter aluminium rod which is inserted into a tungsten sheath with an outer diameter of 1.5 mm. The typical length of the aluminium rod is 7 mm. The beam stopper is stuck onto the upstream side of the polyimide window.

#### 2.2.4. Injector manipulator   

**Figure 3:**
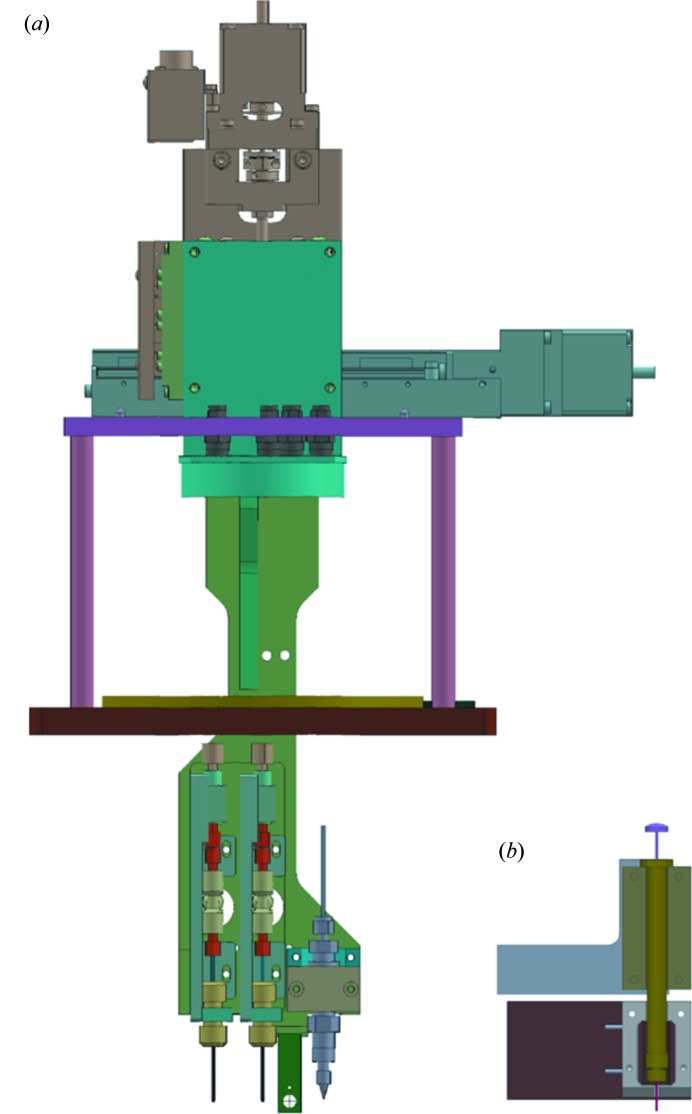
(*a*) Holder for liquid-jet injectors. It is suspended from two motorized stages which translate the injectors in the horizontal and vertical directions. (*b*) Holder for a syringe-pump injector.

Fig. 3 ▸(*a*) shows an injector manipulator for two types of liquid-jet injectors. An injector holder is suspended from motorized linear stages. Three injectors can be installed simultaneously. The holder is equipped with crossed gold wires and a fluorescent screen of cerium-doped yttrium aluminium garnet (Ce:YAG). The crossed wires (0.2 mm in diameter) are used for measuring the intensity distribution of the focused XFEL beam using the knife-edge scan technique. The Ce:YAG screen shows the XFEL position by emitting visible fluorescence induced by X-ray irradiation. The fluorescence is monitored with the microscopes. The injector manipulator is replaced with another one in the case where a syringe-pump injector is installed for highly viscous samples (Fig. 3 ▸ *b*) (Sugahara *et al.*, 2015 ▸).

### 2.3. Injectors   

 In the first SFX experiments at the LCLS, the sample delivery tool was a liquid-jet injector with a gas dynamic virtual nozzle (GDVN), which produces a micrometer-size liquid stream of crystal suspension (DePonte *et al.*, 2008 ▸, 2011 ▸; Weierstall *et al.*, 2012 ▸). After successful demonstrations of the GDVN, other methods were developed for reducing the sample consumption rate. Sierra *et al.* employed the electrospinning technique (Sierra *et al.*, 2012 ▸; Kern *et al.*, 2012 ▸), and were successful in delivering crystal suspensions at flow rates of the order of 10^−1^ µl min^−1^. Another approach for reducing sample consumption is to make a slow flow of crystals dispersed in a highly viscous fluid such as an LCP matrix and a grease-matrix carrier (Liu *et al.*, 2013 ▸; Weierstall *et al.*, 2014 ▸; Sugahara *et al.*, 2015 ▸).

Two types of liquid-jet injectors are employed in DAPHNIS for making a thin stream of soluble protein crystals dispersed in a buffer solution. One is the gas-focusing type (DePonte *et al.*, 2008 ▸, 2011 ▸; Weierstall *et al.*, 2012 ▸; Song *et al.*, 2014 ▸), and the other is used for circulating a sample suspension. These two types can be mounted on one injector holder. The injector for highly viscous samples is installed using another holder.

#### 2.3.1. Liquid-jet injector with a gas-focusing nozzle   

**Figure 4:**
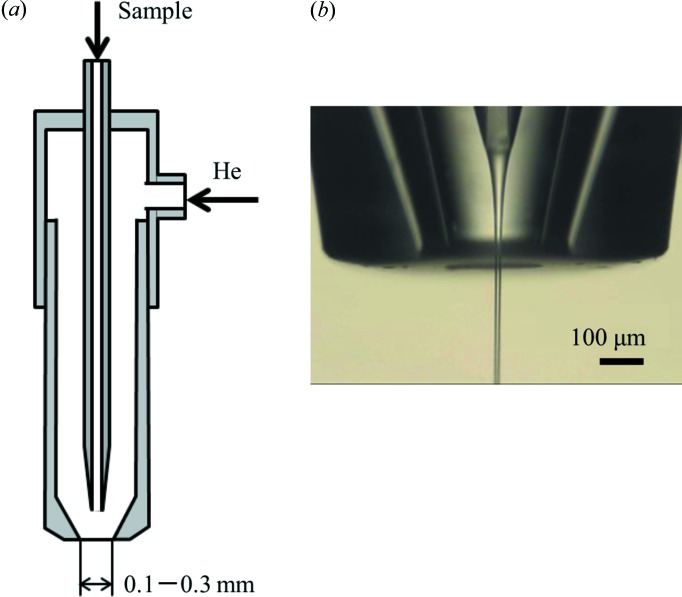
(*a*) Schematic drawing of a liquid-jet injector with a gas focusing nozzle. A He stream is used for focusing a liquid beam ejected from the center capillary. (*b*) Image of the nozzle tip and a liquid beam.

Fig. 4 ▸ shows a schematic drawing of a liquid-jet injector with a gas-focusing nozzle, and a microscope image of the nozzle tip. It has double capillaries to produce the coaxial flow of sample suspension and gas. The inner one with the tapered tip is a channel for sample suspension. Typical nozzle diameters are 50, 75, 100 and 150 µm. The sample suspension is delivered with a hydraulic pump. The gas stream is provided through the outer capillary to reduce the diameter of the sample beam to 4–40 µm. The diameter is varied by the flow rate of liquid and the stagnation pressure of helium gas (see Table S1 in the supporting information).

#### 2.3.2. Liquid-jet injector with sample circulator   

**Figure 5:**
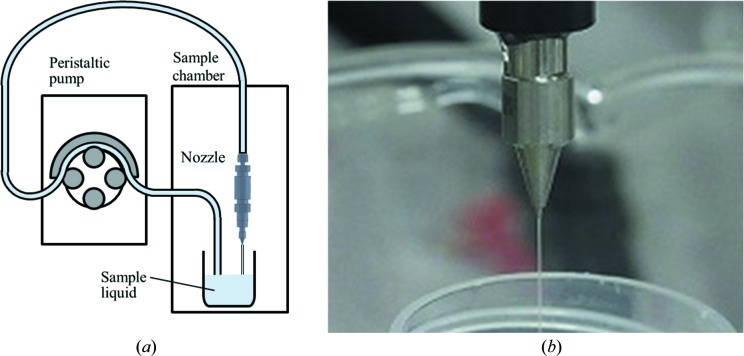
(*a*) Liquid-jet injector with a sample circulator. (*b*) Image of the nozzle tip and a water beam.

The liquid-jet injector with a sample circulation system is schematically shown in Fig. 5(*a*) ▸. The nozzle aperture is 100 or 200 µm in diameter. The liquid-beam size is almost the same as the aperture size. Fig. 5(*b*) ▸ shows an image of the nozzle and a beam of water. The sample is circulated with a peristaltic pump. A typical flow rate with the 100 µm (200 µm) nozzle is 1.5 ml min^−1^ (2.5 ml min^–1^). The circulation system requires ∼5 ml of sample suspension to keep the sample circulating.

#### 2.3.3. Syringe-pump injector for highly viscous samples   

This injector is employed to deliver crystals dispersed in a highly viscous fluid (Sugahara *et al.*, 2015 ▸). A typical flow rate is of the order of 0.1 µl min^−1^. The inner diameter of a standard needle is 110 µm. The temperature of the injector body can be kept constant using a thermoelectric device. The slow flow rate helps to reduce sample consumption; for example, protein consumption can be as small as ∼1 mg in the case of lysozyme. By using a thinner needle with a 50 µm inner diameter, the flow rate can be further reduced to ∼0.03 µl min^−1^.

### 2.4. MPCCD detector with a short working distance   

**Figure 6:**
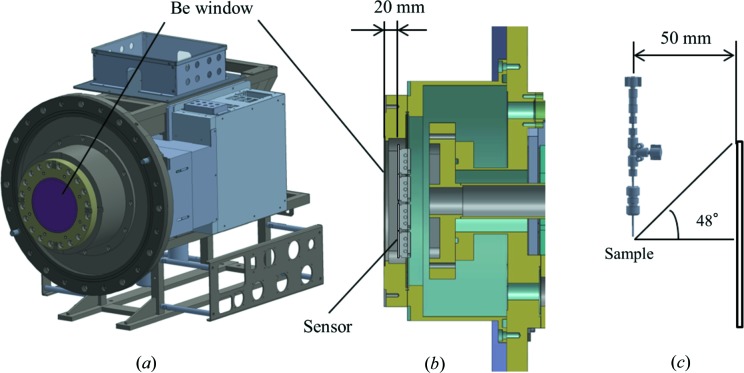
(*a*) MPCCD detector with a short working distance. (*b*) Cross sectional view of the sensor head. (*c*) Positions of the sample and the sensor surface at a sample-to-detector distance of 50 mm.

Specifications and design details of MPCCD detectors have been reported elsewhere (Kameshima *et al.*, 2014 ▸). The detector in DAPHNIS was designed to make the sample-to-sensor distance as small as possible. As shown in Figs. 6 ▸(*a*) and 6(*b*), the sensor housing is sealed with a vacuum-tight beryllium window of 0.45 mm thickness and 115 mm diameter. This window keeps the sensor in vacuum even in ambient-pressure experiments. It also helps to prevent stray light from reaching the sensor. The sensor surface is placed 20 mm behind the Be window. Eight MPCCD modules are tiled to constitute a square sensor area of 110 mm × 110 mm with a square aperture of 3 mm × 3 mm in the center. The distance between the sample and the sensor surface is adjustable between 50 and 100 mm and usually set to be the minimum of 50 mm. In this case the elevation angle from the sample to the center of the upper sensor side is 48° (see Fig. 6 ▸ *c*).

## 3. Experiment   

The SFX experiments were performed at the hard X-ray beamline (BL3) of SACLA (Tono *et al.*, 2013 ▸). The accelerator and photon beamline were tuned to provide an XFEL beam having a center photon energy of 10 keV with a bandwidth of 5 × 10^−3^ (FWHM; full width at half-maximum). The averaged pulse energy at the sample position was estimated at 110 µJ (7 × 10^10^ photons per pulse). The repetition rate was 20 Hz.

The DAPHNIS system was installed in the third experimental hutch (EH3), where the focusing system is permanently stationed. The focal size of the 10 keV beam was measured using the knife-edge scan technique with crossed gold wires. The FWHM at the focal point was 1.5 µm in both the horizontal and vertical directions. The sample chamber was filled with helium gas, the partial pressure of which was kept at greater than 0.9 atm during the diffraction measurement.

A suspension of lysozyme crystals was fed to the interaction point with the gas-focusing liquid-jet injector. The crystals were dispersed in an aqueous solution: 10% (*w*/*v*) sodium chloride and 1.0 *M* sodium acetate (pH 3.0). The number density of the crystals was of the order of 10^9^ cm^−3^. Most of the crystals had similar sizes with long sides of about 1 µm. A microscope image of the crystals is provided in Fig. S1 in the supporting information. The injector provided a 10 µm-diameter stream of the suspension with a flow rate of 0.3 ml min^−1^. The nozzle tip was placed 50 mm away from the sensor surface of the detector.

A sample circulation type injector was also used. A 5 ml suspension of 5 µm crystals was stored in the sample reservoir. The crystal density was 3 × 10^8^ cm^−3^. The suspension was jetted from a nozzle with a 200 µm inner diameter at a flow rate of ∼2.5 ml min^−1^. The diameter of the jet was almost the same as the inner diameter of the nozzle.

Diffraction images were recorded at 20 Hz with the MPCCD detector in a shot-by-shot manner. Bragg spots were indexed by using the *CrystFEL* (version 0.5.2) suite (White *et al.*, 2012 ▸) with *DirAx* (Duisenberg, 1992 ▸) and *MOSFLM* (Powell, 1999 ▸; Leslie, 2006 ▸).

## 4. Results   

In one measurement series using the gas-focusing injector, 45084 images were recorded in about 40 min. Of the 45084 images, 3226 images were able to be processed using the *CrystFEL* software for indexing Bragg spots (7.2% indexing rate). Statistics of the data analysis are summarized in Table 1 ▸. A completeness of 99.8% was achieved at resolution limits of 30.0–2.40 Å. An electron density map was successfully refined at a resolution of 2.4 Å (see Fig. S2 in the supporting information). We obtained higher resolution data by using larger crystals. For example, a 2.0 Å resolution was achieved for crystal sizes of 7–10 µm (Sugahara *et al.*, 2015 ▸). The sample circulation type system also provided a complete data set in a shorter measurement time of about 20 min (see Table 1 ▸). We achieved a resolution of 2.4 Å by using 5694 indexed images.

The present results demonstrate that DAPHNIS provides a complete data set in a reasonable measurement time. In the early SFX studies, ∼10^4^ indexable images are sufficient to construct a complete data set (Kirian *et al.*, 2011 ▸; Boutet *et al.*, 2012 ▸; Weierstall *et al.*, 2014 ▸). Given that the indexing rate is better than 7%, 10^4^ images can be collected in ∼2 h at a repetition rate of 20 Hz. Actually, complete data sets of four kinds of proteins have been obtained within 1 h in our recent experiments (Sugahara *et al.*, 2015 ▸). By refining the experimental conditions, the indexing rate can be enhanced up to about 30%. A higher repetition rate of SACLA (maximum 60 Hz) would also help to reduce the measurement time. Although the measurement time is reasonably short, the sample consumption is relatively high (a few hundreds of milligrams in our case); for example, it was ∼200 mg in the case of 1 µm lysozyme crystals. Sample consumption time can be reduced to ∼1 mg or less by applying highly viscous gel-like carriers with the syringe-pump injector.

## 5. Summary   

The experimental system DAPHNIS has been successfully applied to SFX of proteins. Even 1 µm crystals of lysozyme provide diffraction patterns from which the electron density map was successfully obtained at a resolution of 2.4 Å. It took only 40 min to collect a complete data set. The present results indicate the wide applicability of DAPHNIS to fast structure analysis of proteins of which it has been difficult to synthesize large crystals. Operation under a helium atmosphere is useful in preventing fluid samples from freezing. Three types of injectors are applied to a variety of samples. Owing to the simple and compact structure, DAPHNIS has high adaptability for different applications such as powder X-ray diffraction and X-ray solution scattering. The high adaptability of DAPHNIS can also facilitate future upgrades. For example, it can be easily modified for investigating ultrafast dynamics with a pump-and-probe technique.

## Supplementary Material

S1. Liquid-jet diameters of the gas-focusing nozzle; S2. microscope image of 1-micrometer lysozyme crystals; S3. structure model of lysozyme; Table S1; Figs. S1 and S2. DOI: 10.1107/S1600577515004464/ig5021sup1.pdf

## Figures and Tables

**Table 1:** Data collection and structure refinement statistics for lysozyme

	Liquid jet injector	
	Gas-focusing nozzle	Sample circulation type
Data collection		
Space group	*P*4_3_2_1_2	*P*4_3_2_1_2
Unit-cell parameter		
*a* (Å)	79	79
*b* (Å)	79	79
*c* (Å)	38	38
Wavelength (Å)	1.24	1.24
Number of collected images	45084	20000
Number of indexed patterns	3226	5694
Indexing rate (%)†	7.2	28.8
Number of unique reflections	5038	5085
Resolution range (Å)	30.0–2.40 (2.49–2.40)	30.0–2.40 (2.44–2.40)
Completeness (%)	99.8 (99.8)	100 (100)
*R*_split_ (%)‡	18.9 (33.4)	21.4 (31.4)
CC_1/2_ (%)	92.8 (82.4)	92.2 (81.6)
〈*I*/*σ*(*I*)〉	4.8 (2.8)	4.6 (3.0)
Refinement		
*R*/*R*_free_	19.4/23.0	18.6/22.7
R.m.s. deviations		
Bond lengths (Å)	0.015	0.0042
Bond angles (°)	1.5	0.86
PDB code	3wun	

† Percentage of images that were indexed.

‡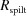 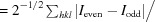 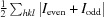.
