# Supplementary material for: Diverse application platform for hard X-ray diffraction in SACLA (DAPHNIS): application to serial protein crystallography using an X-ray free-electron laser
Source: J Synchrotron Radiat. 2015 Apr 16;22(Pt 3):532–7. doi: 10.1107/S1600577515004464 (PMC4817517; doi:10.1107/S1600577515004464)
Supplement: Supplementary file 1 [file s-22-00532-sup1.pdf]

JOURNAL OF SYNCHROTRON RADIATION

Volume 22 (2015)

Supporting information for article:

Diverse application platform for hard X-ray diffraction in SACLA (DAPHNIS): Application to serial protein crystallography using X-ray free electron laser

Kensuke Tono, Eriko Nango, Michihiro Sugahara, Changyong Song, Jaehyun Park, Tomoyuki Tanaka, Rie Tanaka, Yasumasa Joti, Takashi Kameshima, Shun Ono, Takaki Hatsui, Eiichi Mizohata, Mamoru Suzuki, Tatsuro Shimamura, Yoshiki Tanaka, So Iwata and Makina Yabashi

S1. Liquid-jet diameters of the gas-focusing nozzle

A liquid-jet diameter is varied in the range of 4–40 μm with a flow rate of liquid and a stagnation pressure of helium gas. Table S1 shows water-beam diameters at flow rates between 0.06 and 0.32 ml min^{-1} and helium gauge pressures of 0.06, 0.10, and 0.16 MPa.

Table S1 Diameter of a water beam from a capillary with a 50-μm inner diameter.

Water flow rate (ml min^{-1})	Liquid-beam diameter (μm)		
	He 0.06 MPa†	He 0.10 MPa†	He 0.16 MPa†
0.06	unstable	unstable	8
0.08	unstable	unstable	10
0.10	unstable	unstable	11
0.12	unstable	20	13
0.16	32	24	16
0.20	34	28	22
0.24	36	28	22
0.28	38	31	21
0.32	40	32	23

†Gauge pressure.

S2. Microscope image of 1-μm lysozyme crystals

In the diffraction measurement with the gas-focusing liquid-jet injector, the crystals were dispersed in an aqueous solution; 10% (w/v) sodium chloride and 1.0 M sodium acetate (pH 3.0). Figure S1 shows lysozyme crystals in the suspension.

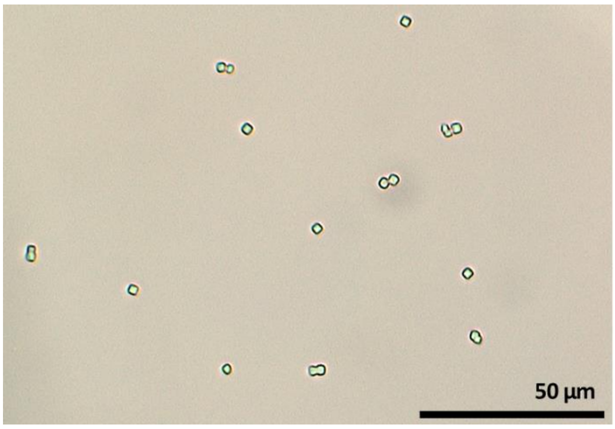

Figure S1 Microscopy image of lysozyme crystals, most of which have long sides of about 1 μm.

S3. Structure model of lysozyme

We obtained 3226 indexed diffraction images of the 1-µm lysozyme crystals which were delivered with the gas-focusing nozzle. The structure model was refined at resolution limits of 30.0–2.40 Å. Figure S2 shows an electron density map at a resolution of 2.4 Å.

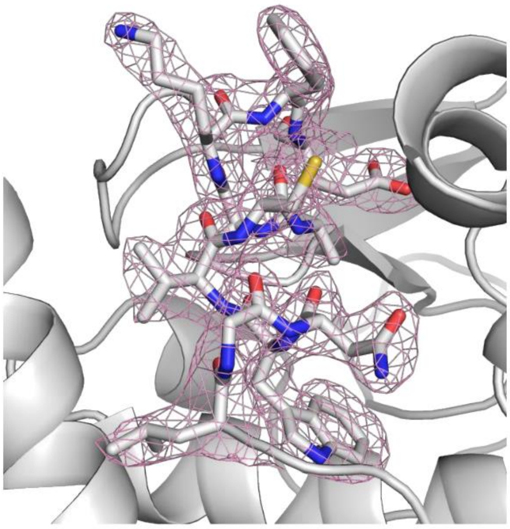

Figure S2 A close-up view of the lysozyme structure with a ($2F_o$–F_c) electron-density map contoured at the 1.0σ level. This figure was drawn with the PyMol program (http://www.pymol.org).
